# Extracellular Hepatitis B Virus RNAs Are Heterogeneous in Length and Circulate as Capsid-Antibody Complexes in Addition to Virions in Chronic Hepatitis B Patients

**DOI:** 10.1128/JVI.00798-18

**Published:** 2018-11-27

**Authors:** Lu Bai, Xiaonan Zhang, Maya Kozlowski, Weixia Li, Min Wu, Jiangxia Liu, Liang Chen, Jiming Zhang, Yuxian Huang, Zhenghong Yuan

**Affiliations:** a Key Laboratory of Medical Molecular Virology, Ministry of Education and Health, School of Basic Medical Sciences, Shanghai Medical College of Fudan University, Shanghai, China; b Shanghai Public Health Clinical Center, Fudan University, Shanghai, China; c Department of Infectious Diseases, Huashan Hospital, Fudan University, Shanghai, China; University of Southern California

**Keywords:** capsid, capsid-antibody complex, extracellular HBV RNA, hepatitis B virus

## Abstract

Extracellular HBV RNA has been detected in both HBV-replicating cell culture media and sera from chronic hepatitis B (CHB) patients, but its exact origin and composition remain controversial. Here, we demonstrated that extracellular HBV RNA species were of heterogeneous lengths, ranging from the length of pregenomic RNA to a few hundred nucleotides. In cell models, these RNAs were predominantly associated with naked capsids, although virions also harbored a minority of them. Moreover, HBV RNAs in hepatitis B patients’ blood circulation were localized in unenveloped capsids in the form of capsid-antibody complexes (CACs) and in virions. Furthermore, we showed that extracellular HBV RNAs could serve as the template for viral DNA synthesis. In conclusion, extracellular HBV RNAs mainly consist of pgRNA or the pgRNA species degraded by the RNase H domain of the polymerase in the process of viral DNA synthesis and circulate as CACs and virions. Their presence in blood circulation of CHB patients may be exploited to develop novel biomarkers for HBV persistence.

**IMPORTANCE** Although increasing evidence suggests the presence of extracellular HBV RNA species, their origin and molecular forms are still under debate. In addition to the infectious virions, HBV is known to secrete several species of incomplete viral particles, including hepatitis B surface antigen (HBsAg) particles, naked capsids, and empty virions, during its replication cycle. Here, we demonstrated that extracellular HBV RNAs were associated with naked capsids and virions in HepAD38 cells. Interestingly, we found that unenveloped capsids circulate in the blood of hepatitis B patients in the form of CACs and, together with virions, serve as vehicles carrying these RNA molecules. Moreover, extracellular HBV RNAs are heterogeneous in length and represent either pregenomic RNA (pgRNA) or products of incomplete reverse transcription during viral replication. These findings provide a conceptual basis for further application of extracellular RNA species as novel biomarkers for HBV persistence.

## Abstract (precis)

Although increasing evidence suggests the presence of extracellular HBV RNA species, their origin and molecular forms are still under debate. In addition to the infectious virions, HBV is known to secrete several species of incomplete viral particles, including hepatitis B surface antigen (HBsAg) particles, naked capsids, and empty virions, during its replication cycle. Here, we demonstrated that extracellular HBV RNAs were associated with naked capsids and virions in HepAD38 cells. Interestingly, we found that unenveloped capsids circulate in the blood of hepatitis B patients in the form of CACs and, together with virions, serve as vehicles carrying these RNA molecules. Moreover, extracellular HBV RNAs are heterogeneous in length and represent either pregenomic RNA (pgRNA) or products of incomplete reverse transcription during viral replication. These findings provide a conceptual basis for further application of extracellular RNA species as novel biomarkers for HBV persistence.

## INTRODUCTION

Hepatitis B virus (HBV) is still a major global health problem, with an estimated 257 million people worldwide that are chronically infected with HBV (1). HBV, together with duck hepatitis B virus (DHBV) and several other related animal viruses, belongs to the *Hepadnaviridae* family (2). The HBV virion is comprised of an outer envelope and an inner icosahedral nucleocapsid (NC) assembled by 240 copies of core protein (HBc) and packaged with a 3.2-kb partially double-stranded circular DNA genome (3–8). In addition to DNA-containing virions, a large amount of incomplete viral particles, such as hepatitis B surface antigen (HBsAg) particles, empty virions, and naked capsids, can also be released from cells in the process of virus replication (9). Subviral HBsAg particles are spherical or rodlike and are present in vast excess over virions in sera of CHB patients (2). Empty virions share the same structure as DNA-containing virions but are devoid of nucleic acids (10–14). Naked capsids, which exit cells via a route different from that of virions (15–17), have the same structure as NCs but are either empty or filled with viral RNA and immature viral DNA (7, 11, 18–20).

In NC, pgRNA undergoes reverse transcription into minus-strand DNA, followed by plus-strand DNA synthesis (2, 21–24). Intracellular NCs can be packaged with viral nucleic acids at all levels of maturation, including pgRNA, nascent minus-strand DNA, minus-strand DNA-RNA hybrids, and relaxed circular DNA (RC DNA) or double-stranded linear DNA (DSL DNA) (5, 7). Only the NCs with relatively mature viral DNA (RC or DSL DNA) are enveloped and secreted as virions. HBV replicating cells can release empty core particles assembled from HBc proteins and NCs that contain various species of replicative intermediate nucleic acids into the culture supernatant. However, while free naked capsids could be readily detected *in vitro* (7, 11, 18–20), they are hardly found in the blood of HBV-infected patients (17, 25, 26).

Although extracellular HBV RNA was detected in both *in vitro* cell culture systems and in clinical serum samples, its origin and composition remain controversial. It was proposed that extracellular HBV RNA represents pgRNA localized in virions (27). However, HBV spliced RNA and HBx RNA were also detected in culture supernatant of HBV stably replicating cells as well as in sera of CHB patients (28, 29). In addition, extracellular HBV RNA was also suggested to originate from damaged liver cells (30), naked capsids, or exosomes (11, 29). Hence, these extracellular RNA molecules have never been conclusively characterized. Here, we demonstrate that extracellular HBV RNAs are heterogeneous in length, ranging from full-length pgRNA (3.5 kilonucleotides [knt]) to RNA fragments with merely several hundred nucleotides. These RNA molecules represent 3′ receding pgRNA fragments that have not been completely reverse transcribed to DNA and pgRNA fragments hydrolyzed by the RNase H domain of polymerase in the process of viral replication. More importantly, extracellular HBV RNAs are localized in naked capsids and in virions in culture supernatants of HBV replicating cells and also circulate as CACs and virions in blood of hepatitis B patients.

## RESULTS

### Extracellular HBV RNAs are heterogeneous in length and predominantly integral to naked capsids instead of virions in HepAD38 cell culture supernatant.

**FIG 1:**
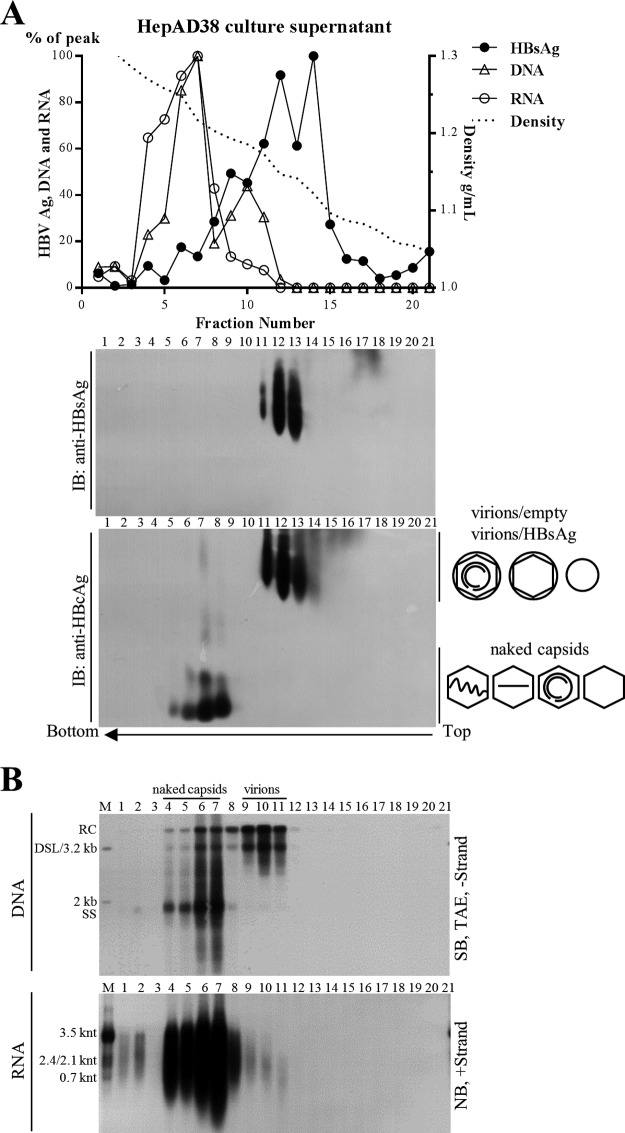
Sucrose gradient separation and analysis of viral particles from HepAD38 cell culture supernatant. (A) Distribution of hepatitis B viral particle-associated antigens and DNA/RNA in sucrose gradient. Viral particles prepared from HepAD38 cell culture supernatant (via PEG 8000 precipitation) were layered over a 10% to 60% (wt/wt) sucrose gradient for ultracentrifugation separation. Fractions were collected from top to bottom, and HBsAg level was analyzed by enzyme-linked immunosorbent assay (ELISA). HBsAg and viral DNA and RNA (quantified from gray density of bands in panel B) signals and sucrose density were plotted together. Viral particles were first resolved by native agarose gel electrophoresis, followed by immunoblotting (IB) of HBV envelope and core proteins with anti-HBsAg and anti-HBcAg antibodies. (B) Detection of viral DNA/RNA by Southern or Northern blotting. Total viral nucleic acids were extracted by the SDS-proteinase K method, and viral DNA (extracted from one-tenth of the samples used for Northern blotting) and RNA (treated with DNase I) were detected by Southern and Northern blot analyses with minus- or plus-strand-specific riboprobes, respectively. Symbols of HBsAg particles, empty virions (without nucleic acid), virions (with RC DNA), and naked capsids (empty or with nucleic acids) are depicted on the lower right side of panel A. Blank, no nucleic acids; two centered and gapped circles, RC DNA; straight line, SS DNA; wavy lines, pgRNA; M, markers (50 pg of 1-kb, 2-kb, and 3.2-kb DNA fragments released from plasmids as the DNA ladder or total RNA extracted from HepAD38 cells as the RNA ladder).

To ascertain the origin of extracellular HBV RNA, we first examined viral particles prepared from culture medium of an *in vitro* HBV stably transduced cell line. A human hepatoma HepAD38 cell line was used in this study, as it sustains vigorous HBV replication under the control of a tetracycline-repressible cytomegalovirus (CMV) promoter (31). Total viral particles were concentrated and centrifuged over a 10% to 60% (wt/wt) sucrose gradient. Most of the subviral HBsAg particles, virions, and empty virions were detected between fractions 9 to 14 (Fig. 1A, upper and middle). Naked capsids, detected only by anti-HBcAg and not by anti-HBsAg antibodies, settled in fractions 5 to 8 (Fig. 1A, middle and lower). The majority of viral nucleic acids were detected in fractions between 4 and 11 (Fig. 1B, upper), which coincided with the fractions containing virions (fractions 9 to 11), naked capsids (fractions 4 to 7), and the mixture of these particles (fraction 8). Consistent with previous observations, HBV virions are packed with mature viral DNA (RC or DSL DNA), while naked capsids contain both immature single-stranded DNA (SS DNA) and mature viral DNA (Fig. 1B, upper). Moreover, Northern blot results showed that most of the HBV RNA was detected in the naked capsids (Fig. 1B, lower, fractions 4 to 7), whereas only a very small amount was associated with virions (Fig. 1B, lower, fractions 9 to 11). HBV RNA detected in naked capsids ranged from the full length of pgRNA down to a few hundred nucleotides (shorter than the HBx mRNA [0.7 knt]). Moreover, RNA molecules within virions were much shorter than those within naked capsids. We excluded the possibility of artifacts generated by the SDS-proteinase K extraction method, as a similar RNA blot pattern was obtained using a TRIzol reagent to extract both intracellular nucleocapsid-associated and extracellular HBV RNA (not shown). Furthermore, quantification of viral RNA extracted by either the SDS-proteinase K method or TRIzol reagent produced a very similar copy number, except that the TRIzol reagent is known to preferentially extract RNA rather than DNA (not shown). Moreover, the RNA signal detected by Northern blotting could not be attributed to DNA fragments generated by DNase I treatment, which would reduce DNA to below the detection limit of the hybridization method (not shown). Furthermore, the RNA signal could be completely removed by an additional RNase A treatment (not shown).

**FIG 2:**
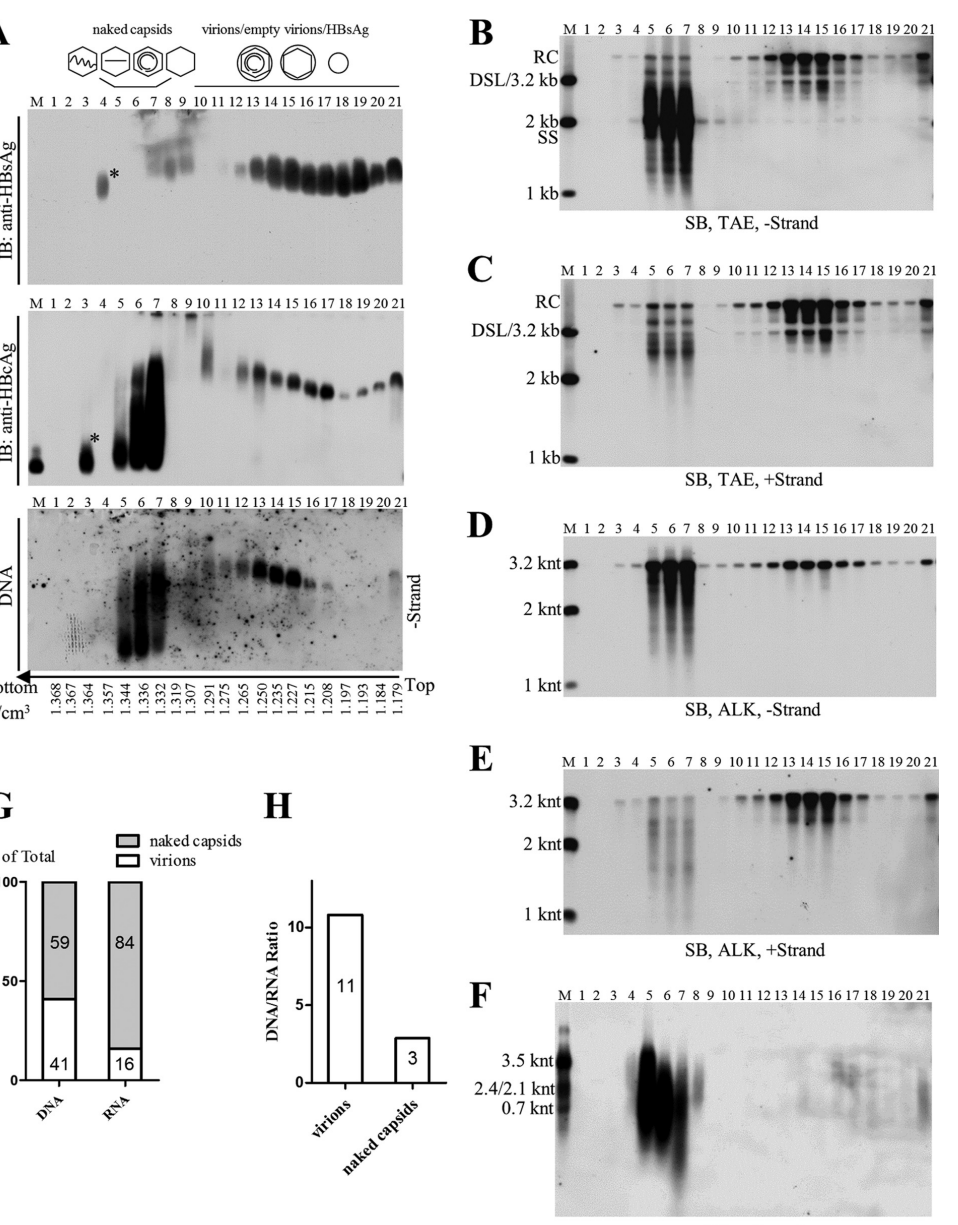
CsCl density gradient separation and analysis of viral particles from HepAD38 cell culture supernatant. (A) Native agarose gel analysis of viral particles. Culture supernatant of HepAD38 cells was concentrated (via ultrafiltration) and fractionated by CsCl density gradient centrifugation (3 ml of 1.18 g/cm^3^ CsCl solution in the upper layer and 1.9 ml of 1.33 g/cm^3^ CsCl solution in the lower layer). Viral particles in each fraction were resolved by native agarose gel electrophoresis, followed by detection of viral antigens with anti-HBsAg and anti-HBcAg antibodies and viral DNA by hybridization with minus-strand-specific riboprobe. (B to F) Southern and Northern blot detection of viral nucleic acids. Viral DNAs were separated by electrophoresis through Tris-acetate-EDTA (TAE) or alkaline (ALK) agarose gel for Southern blotting with minus- or plus-strand-specific riboprobes. Viral RNA was obtained by treatment with total nucleic acids with DNase I and separated by formaldehyde-MOPS agarose gel, followed by Northern blotting. (G) Quantification of viral DNA and RNA in naked capsids or virions. Fractions containing naked capsids (fractions 3 to 7) or virions (fractions 10 to 21) were pooled, and viral DNA and RNA were quantified by PCR. (H) DNA and RNA ratios in naked capsids and virions calculated based on quantitative results. Asterisks indicate unknown high-density viral particles detected by anti-HBcAg or anti-HBsAg antibodies but devoid of any HBV-specific nucleic acids. M, markers (E. coli-derived HBV capsids or DNA and RNA ladders as described in the legend to Fig. 1).

To confirm the above-described results and to better separate naked capsids from HBV virions, isopycnic CsCl gradient ultracentrifugation was employed. Naked capsids were observed mainly in fractions 5 to 7, with densities ranging from 1.33 to 1.34 g/cm^3^ (Fig. 2A). The smearing bands of naked capsids were likely caused by high concentrations of CsCl salt, as fractionation of naked capsids in a 1.18-g/cm^3^ CsCl solution produced single bands. Virions, detected by both anti-HBcAg and anti-HBsAg antibodies (Fig. 2A, upper and middle), were packaged with viral DNA (Fig. 2A, lower) and settled in fractions 13 to 15, with densities ranging from 1.23 to 1.25 g/cm^3^. In agreement with the results shown in Fig. 1, HBV virions contained only the mature viral DNA (RC or DSL DNA), while naked capsids contained viral DNA replicative intermediates that ranged from the nascent minus-strand DNA to mature viral DNA (Fig. 2B and C). The lengths of viral minus- and plus-strand DNA in naked capsids and virions were determined by alkaline agarose gel electrophoresis analysis, a condition where denatured single-stranded DNA molecules migrate according to their lengths. In contrast to the complete minus- and mostly complete plus-strand DNA (closed to 3.2 knt) in virions, in naked capsids the minus-strand DNA and the plus-strand DNA can be both complete and incomplete (shorter than 3.2 knt) (Fig. 2D and E). Moreover, the length of HBV RNAs within naked capsids still ranged from 3.5 knt of pgRNA to shorter than the 0.7 knt of HBx mRNA. Full-length pgRNA accounted for only 10% of total RNA signal detected by Northern blotting (quantified from gray density of bands shown in Fig. 2F). In contrast, HBV RNA species in virions are relatively shorter and barely detectable. In addition, we also determined viral DNA and RNA copy numbers in pooled naked capsids (fractions 3 to 7) and virions (fractions 10 to 21) by quantitative PCR. Quantification results showed that viral DNA in naked capsids and in virions accounted for about 60% and 40%, respectively, of total viral DNA signal in the HepAD38 cell culture supernatant (Fig. 2G). More importantly, 84% of the HBV RNA was associated with naked capsids, while merely 16% was detected within virions (Fig. 2G). Additionally, the DNA/RNA ratio was 11 in virions and 3 in naked capsids (Fig. 2H), suggesting that more HBV RNA is present in naked capsids.

### Extracellular HBV RNAs and immature viral DNA are detected in sera from CHB patients.

**FIG 3:**
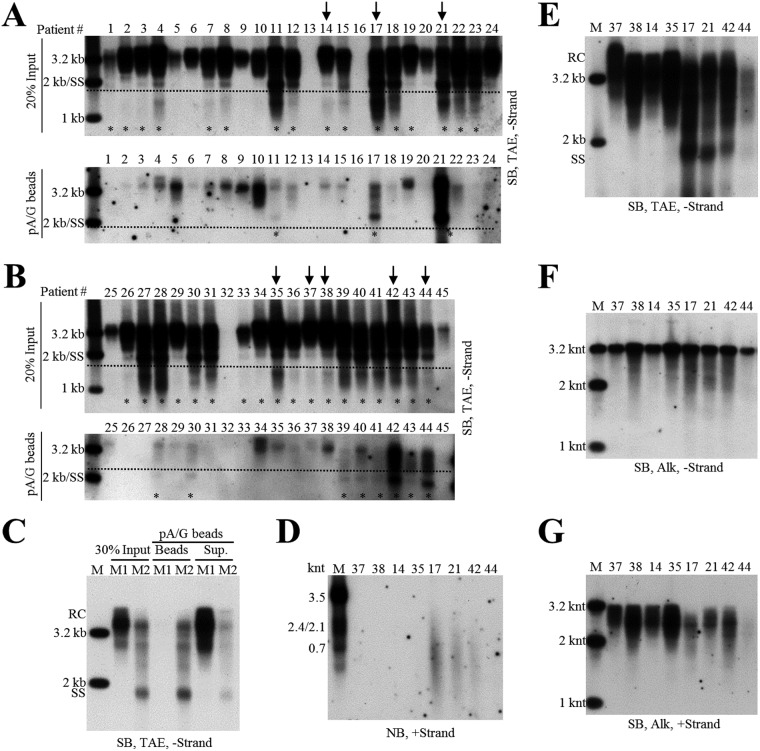
Characterization of HBV DNA and RNA in sera of CHB patients. (A and B) Analyses of serum viral DNA from CHB patients by Southern blotting. Viral DNA was extracted from serum samples obtained from forty-five chronic hepatitis B patients (20% of input sample used for protein A/G agarose beads pulldown) and subjected to Southern blot analysis. Alternatively, these samples were first incubated with protein A/G agarose beads, and then viral DNA in the pulldown mixtures was analyzed by Southern blotting. Serum samples selected for further examining are marked with arrows, and samples with SS DNA detection are labeled with asterisks. (C) Protein A/G agarose bead pulldown of viral particles. Sera (25 μl each) from CHB patients 37, 38, 14, and 35 (M1, mixture one) or from patients 17, 21, 42, and 44 (M2, mixture two) were pooled and incubated with protein A/G agarose beads. Viral DNA in input sera, protein A/G bead pulldown mixtures (beads), and the remaining supernatants (sup.) were extracted and subjected to Southern blot analysis. (D) Northern blot detection of serum viral RNA from patients 37, 38, 14, 35, 17, 21, 42, and 44. Total RNA were extracted from serum samples by TRIzol reagent and treated with DNase I before Northern blot analysis. (E to G) Southern blot analyses of viral DNA from selected samples. Viral DNA was separated by electrophoresis through TAE or alkaline agarose gels, followed by Southern blot detection with the indicated riboprobes.

Employing the HepAD38 cell culture system, we demonstrated the presence of extracellular HBV RNAs and immature and mature viral DNA packaged in both the naked capsids and virions. Interestingly, Southern blot analyses showed that SS DNA could also be observed in serum samples from some CHB patients. We speculated that SS DNA in circulation would be carried by capsid particles that were released by HBV-infected hepatocytes into patients’ bloodstreams. However, we reasoned that due to strong immunogenicity of naked capsids (32, 33), it would be difficult to detect them as free particles; rather, they would form complexes with specific anti-HBcAg antibodies and therefore circulate as antigen-antibody complexes (25, 32–34). To entertain this possibility, we then used protein A/G agarose beads to pull down the immune complexes. Forty-five serum samples obtained from CHB patients, with HBV DNA titers higher than 10^7^ IU per ml, were examined for the presence of particles containing SS DNA by a combination of protein A/G agarose bead pulldown assay and Southern blot analysis (Fig. 3A and B). SS DNA was detected, albeit to a different extent, in 34 serum samples (Fig. 3A and B, upper). The particles containing SS DNA were pulled down by protein A/G agarose beads from 11 out of the 34 samples (Fig. 3A and B, lower). Patient sera negative for SS DNA (patients 37, 38, 14, and 35) or positive for SS DNA (patients 17, 21, 42, and 44), as determined by the protein A/G agarose bead pulldown experiments, were selected for further studies (Fig. 3C).

Northern blot analyses showed that HBV RNA was only detected in serum samples from patients 17, 21, and 42 (Fig. 3D). Moreover, total viral DNA was analyzed by Southern blotting, and SS DNA was readily observed in serum samples from patients 17, 21, and 42 (Fig. 3E). We also analyzed the lengths of DNA minus and plus strands in patients’ sera. Despite the finding that most minus-strand DNA was complete, a small amount of viral DNA (that of patients 38, 35, 17, 21, and 42) was shorter than 3.2 knt (Fig. 3F). Compared with viral minus-strand DNA, the length of plus-strand DNA, particularly in sera from patients 17, 21, and 42, was more variable, ranging from shorter than 2 knt to ∼3.2 knt (Fig. 3G).

### Naked capsids form CACs with anti-HBcAg antibody in blood of CHB patients.

**FIG 4:**
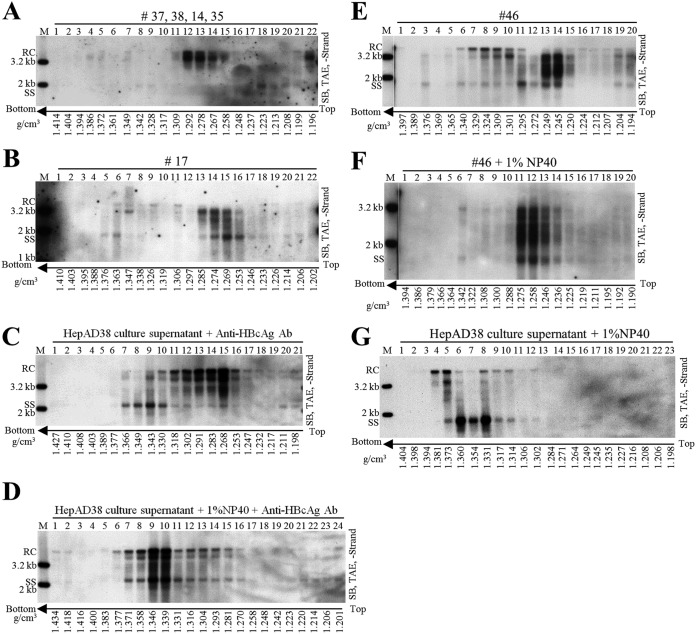
CsCl density gradient analysis of hepatitis B viral particles. (A and B) CsCl density gradient analysis of viral particles in patient sera. One hundred-microliter volumes of serum mixture from patients 37, 38, 14, and 35 (25 μl each) and 100 μl serum from patient 17 were separated by CsCl density gradient centrifugation (2 ml of 1.18 g/cm^3^ CsCl solution in the upper layer and 2.9 ml of 1.33 g/cm^3^ CsCl solution in the lower layer). Viral DNA in each fraction was extracted and detected by Southern blotting. (C to G) CsCl density gradient analysis of viral particles treated with detergent or anti-HBcAg antibody (Ab). Concentrated HepAD38 cell culture supernatant (250 μl each) (via ultrafiltration) was either mixed with anti-HBcAg antibody (10 μl) followed by incubation without (C) or with NP-40 (final concentration, 1%) (D) for 1 h at room temperature and 4 h on ice or treated with only NP-40 (G) and then fractionated by CsCl density gradient ultracentrifugation. Sera from CHB patient 46 either left untreated (E) or treated with NP-40 (final concentration, 1%) (F) were fractionated by CsCl density gradient ultracentrifugation. Viral DNA in each fraction was extracted and subjected to Southern blot analyses.

We showed that particles containing SS DNA were present in CHB patients’ sera. To further examine these particles, we used CsCl density gradient centrifugation to fractionate a serum mixture from patients 37, 38, 14, and 35. In agreement with our earlier results (Fig. 2A, lower, fractions 13 to 15, and B) and previous reports, HBV virions, with the characteristic mature viral DNA (RC or DSL DNA), were detected in fractions 12 to 14 with densities between 1.26 and 1.29 g/cm^3^ (Fig. 4A) (2). Careful inspection of the blots revealed that SS DNA could be detected, albeit at very low level, in fractions 8 and 9, with densities from 1.33 to 1.34 g/cm^3^, and in fractions 18 to 21, with densities from 1.20 to 1.23 g/cm^3^ (Fig. 4A). In contrast, CsCl density gradient separation of viral particles from serum of patient 17 showed a mixture of mature and immature viral DNA species. As SS DNA was detected at densities ranging from 1.37 to 1.20 g/cm^3^ (Fig. 4B), no distinct viral DNA (mature RC or DSL DNA) specific to virions could be identified at densities between 1.27 and 1.29 g/cm^3^. Similar results were obtained using CsCl density gradient fractionation of sera from patient 21 (not shown) and patient 46 (Fig. 4E).

We hypothesized that naked capsids could be released into blood circulation of CHB patients but were bound to specific antibodies. As SS DNA was detected in both high- and lower-density regions in CsCl gradient (Fig. 4B and E), we envisaged that the binding with specific antibodies led to a change of capsids’ buoyant density. To test this, anti-HBcAg antibody was mixed with HepAD38 cell culture supernatant to mimic the postulated CACs in serum samples. The results demonstrated that in contrast to SS DNA from naked capsids, distributed to three fractions at densities between 1.33 and 1.34 g/cm^3^ (Fig. 2A, lower, and B), the mixture of naked capsids and CACs (SS DNA) was distributed more widely and could be detected in the lower density region (1.25 to 1.32 g/cm^3^) (Fig. 4C, fractions 11 to 16). Similarly, intracellular capsids from HepAD38 cells were incubated with anti-HBcAg antibody, and a density shift of CACs to a lower-density region was also observed (not shown). To further confirm the lower density of CACs, NCs in virions secreted to HepAD38 cell culture supernatant were treated with NP-40 and mixed with anti-HBcAg antibody. CsCl fractionation showed that naked capsids and virion-derived NCs have become a homogenous mixture banding at densities from 1.37 to 1.27 g/cm^3^ (Fig. 4D). Likewise, virion-derived NCs, obtained by treatment of serum sample from patient 46 with NP-40 bound with antibody, further formed new homogeneous CACs that settled at densities between 1.23 and 1.27 g/cm^3^ (Fig. 4E versus F). However, NP-40 treatment alone did not produce a homogeneous mixture of naked capsids and virion-derived NCs, as these two particles still settled at distinct density regions with their characteristic viral DNA content (Fig. 4G). On the other hand, DNA molecules in the two types of capsids still banded at densities between 1.38 and 1.31 g/cm^3^, further confirming that CACs have relatively lighter density (Fig. 4G).

**FIG 5:**
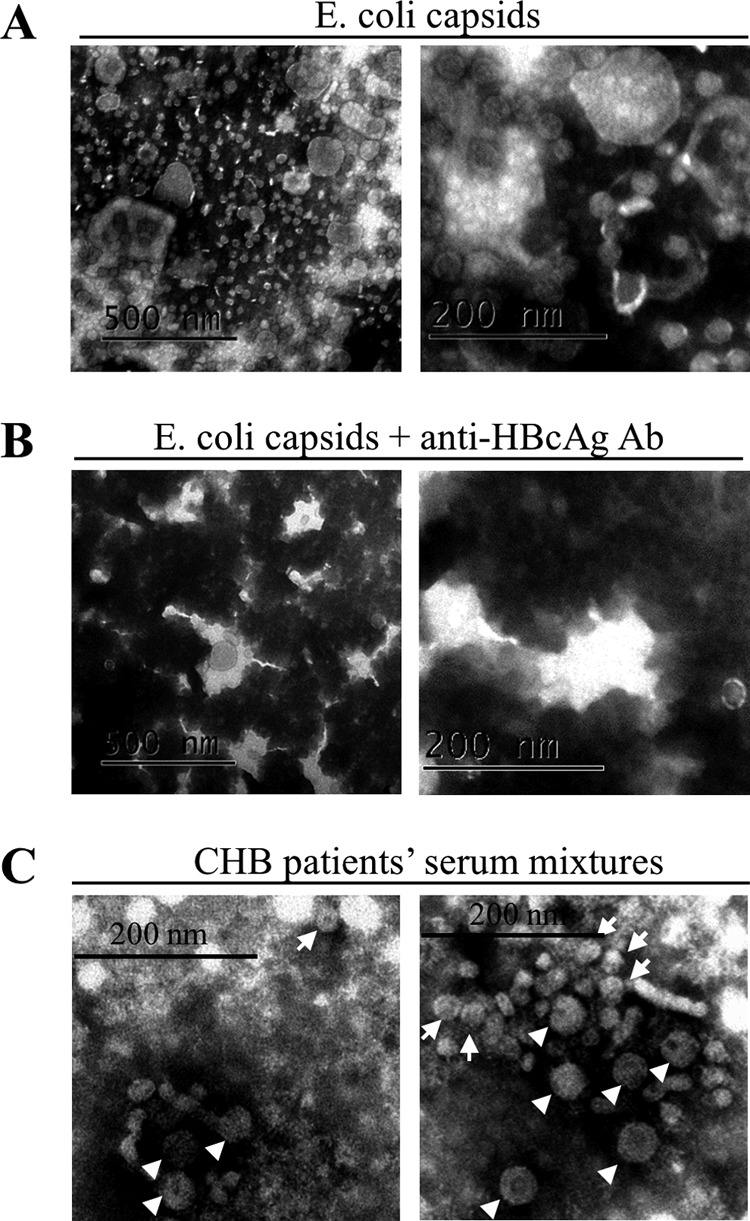
EM analysis of hepatitis B viral particles. (A and B) EM of E. coli-derived HBV capsids incubated without or with anti-HBcAg antibody. (C) EM of viral particles prepared from sera of CHB patients. Serum mixtures (obtained from patients 11, 22, 23, 27, 28, 30, and 41) depleted of HBsAg particles were negatively stained and examined with an electron microscope. The 42-nm HBV virions (arrowhead) and 27-nm naked capsids (arrow) are indicated, while the smaller 22-nm rods and spheres of HBsAg particles could also be observed but are not pointed out. Scale bars indicate 200 nm or 500 nm.

Alternatively, the appearance of a homogenous mixture of virion-derived NCs and naked capsids (Fig. 4D and F) suggests the formation of higher-order antibody-mediated complexes of capsids. For instance, the complexes might not represent individual antibody-coated capsid particles but rather big CACs consisting of several capsid particles interconnected by antibodies. To verify whether intercapsid immune complexes exist, anti-HBcAg antibody was added to the purified HBV capsids expressed by Escherichia coli, and this mixture was examined by an electron microscope. E. coli-derived capsids were scattered as separate, distinct particles (Fig. 5A). However, addition of antibody caused capsids to aggregate into clusters, making them too thick to be properly stained (Fig. 5B). Despite this, a few capsids, which might not have been bound by antibodies or might have been associated with antibodies but did not form intercapsid antibody complexes, could be observed by electron microscopy (EM) (Fig. 5B).

We then examined CACs in serum samples from CHB patients by EM. Sera from patients 11, 17, 21, 22, 23, 27, 28, 30, and 41, positive for SS DNA, were combined. Serum mixtures, with diminished HBsAg particles by centrifugation through a 20% and 45% (wt/wt) sucrose cushion, were examined by EM. The 27-nm capsid particles or CACs were visible (Fig. 5C, arrow) along with the 42-nm HBV virions (Fig. 5C, arrowheads) and the 22-nm spheres and rods of residual HBsAg particles (not indicated). However, the picture was not clear enough for us to conclusively determine if capsids were connected by or bound with antibodies, as described for unrelated virus in *in vitro* experiments (35). In addition, it is possible that some of the CACs are not visible by EM, as the complexes maybe too thick to gain clear contrast between lightly and heavily stained areas (Fig. 5B).

**FIG 6:**
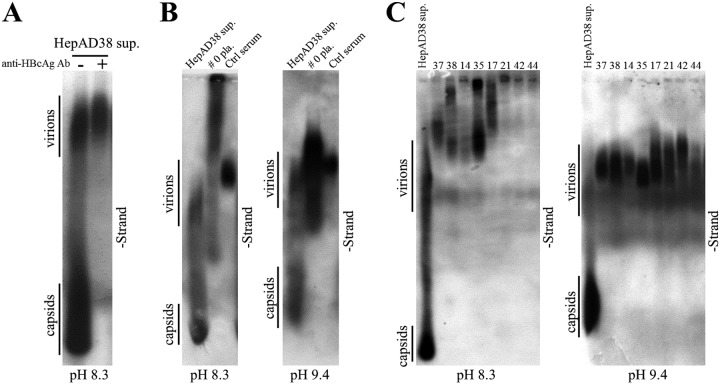
Native agarose gel analysis of viral particles in sera from hepatitis B patients. (A) Native agarose gel analysis of viral particles from HepAD38 cell culture supernatant. Ten microliters of HepAD38 cell culture supernatant (concentrated by ultrafiltration) incubated with or without anti-HBcAg antibody was resolved by native (TAE) agarose gel (0.8%) electrophoresis, followed by hybridization with minus-strand-specific riboprobe. (B and C) Native agarose gel analysis of viral particles from serum samples of hepatitis B patient in buffer with different pH values. Ten microliters of concentrated HepAD38 cell culture supernatant, plasma sample of patient 0 (not concentrated), and serum of a chronic hepatitis B carrier without liver inflammation (ctrl serum) were loaded into agarose gels prepared in TAE buffer (pH 8.3) (B, left) or Dunn carbonate buffer (10 mM NaCHO_3_, 3 mM Na_2_CO_3_, pH 9.4) (B, right) and separated overnight. Viral particle-associated DNA was detected by hybridization with specific riboprobe. Sera from patients 37, 38, 14, 35, 17, 21, 42, and 44 (10 μl each) were resolved by electrophoresis through 0.7% high-strength agarose (type IV agarose used for pulsed-field gel electrophoresis) gels prepared in TAE (C, left) or carbonate buffer (C, right), followed by probe hybridization.

Lastly, CACs might be heterogeneous, having different molecular sizes and isoelectric points (pI) in hepatitis B patients’ blood circulation. *In vitro* binding of naked capsids derived from HepAD38 cell culture supernatant with anti-HBcAg antibody changed their electrophoretic behavior and made them unable to enter the TAE-agarose gel (Fig. 6A). Moreover, viral particles from sera of patients 0, 37, 38, 14, 35, 17, 21, 42, and 44 could not enter agarose gels prepared in TAE buffer. However, in buffer with higher pH value (10 mM NaCHO_3_, 3 mM Na_2_CO_3_, pH 9.4), they appeared as smearing bands on blots (Fig. 6B and C). Hence, the irregular electrophoretic behavior of these viral particles may result from changes in molecular size and/or pI value of capsid particles (pI  4.4) following their association with specific immunoglobulin G (or other types of antibodies) having different pI values (pI of human IgG may range from 6.5 to 9.5) (36–39).

### Circulating HBV RNAs are of heterogeneous lengths and associated with CACs and virions in hepatitis B patient’s plasma.

**FIG 7:**
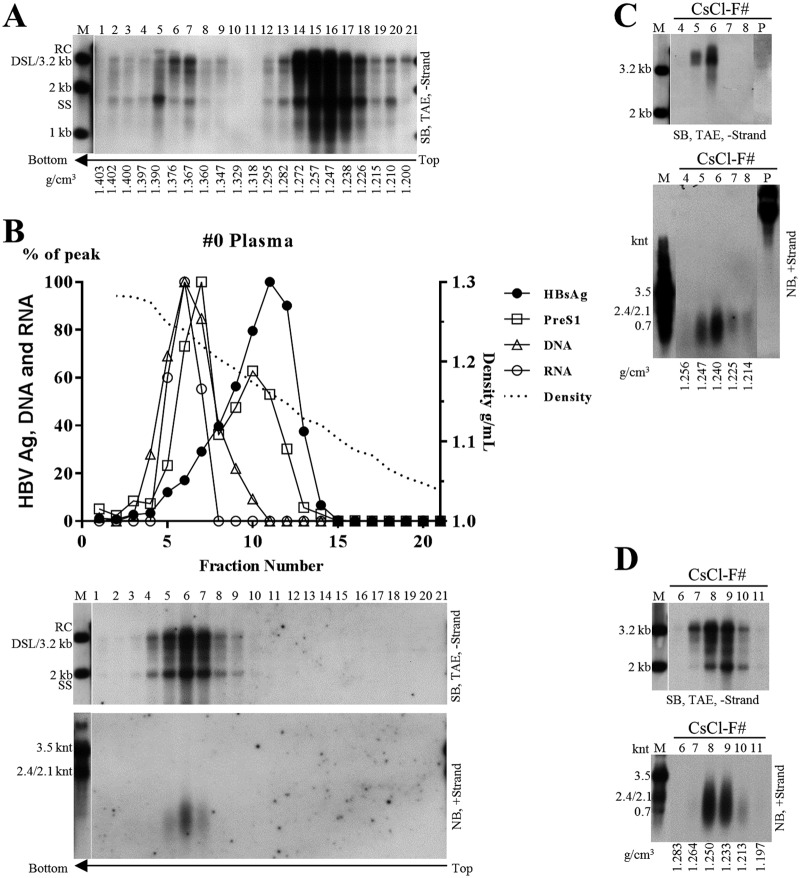
Characterization of nucleic acid content within viral particles in plasma sample from patient 0. (A) CsCl density gradient analysis of plasma sample. Plasma from patient 0 was added directly with CsCl salt to a concentration of 21% (wt/wt) or 34% (wt/wt). Two milliliters of the 21% CsCl-plasma mixture was underlayered with 2.9 ml 34% CsCl-plasma mixture, followed by ultracentrifugation. Viral DNA from each fraction was extracted and subjected to Southern blot analysis. (B) Sucrose gradient analysis of concentrated plasma sample. Five hundred microliters of concentrated plasma sample (via ultracentrifugation through a 20% sucrose cushion) was fractionated in a 10% to 60% (wt/wt) sucrose gradient. PreS1 and HBsAg levels were determined by ELISA. Viral DNA and RNA were detected by Southern and Northern blotting with minus- or plus-strand-specific riboprobes. HBsAg, PreS1, and viral DNA and RNA (quantified from gray density of viral DNA/RNA bands, middle and lower) signals and sucrose density were plotted together. (C) Analysis of concentrated plasma sample with lower CsCl density gradient centrifugation. Two hundred fifty microliters of concentrated plasma sample was mixed with 2.2 ml TNE buffer and 2.45 ml of 37% (wt/wt) CsCl-TNE buffer (resulting in a homogenous CsCl solution with density of about 1.18 g/cm^3^), followed by ultracentrifugation. DNA in viral particle pellets (lane P) stuck to the sidewall of centrifugation tubes and was recovered by digesting with SDS-proteinase K solution. Viral DNA and RNA were subjected to Southern and Northern blot analyses. (D) Analysis of concentrated plasma sample with higher level of CsCl density gradient centrifugation. Two hundred fifty microliters of concentrated plasma sample was mixed with 1 ml of TNE buffer and 1.25 ml of 37% (wt/wt) CsCl-TNE buffer and underlayered with 2.4 ml of 27% (wt/wt) (1.25 g/cm^3^) CsCl-TNE solution, followed by ultracentrifugation. HBV DNA and RNA was detected by Southern and Northern blotting.

To characterize HBV RNAs circulating in CHB patients’ sera, a plasma sample from patient 0 was studied. Similar to results obtained for patients 17, 21, and 46 (Fig. 4B and E and not shown), viral DNA in the plasma sample of patient 0 was detected in a broad density range in CsCl gradient and no distinct bands specific to HBV virions or naked capsids could be identified, indicating the presence of a mixture of virions and CACs (Fig. 7A).

Furthermore, viral particles were pelleted through a 20% sucrose cushion and separated in a sucrose gradient. HBsAg was detected in fractions 5 to 14, peaking at fraction 11. The PreS1 antigen was found in fractions 5 to 12 with the peak at fractions 7 and 10, indicating its presence in HBsAg particles and HBV virions (Fig. 7B, upper). Viral DNA, representing a combination of both mature and immature viral DNA, was detected in fractions 4 to 9 (Fig. 7B, middle), suggesting the localization of CACs and virions in these fractions. HBV RNA was detected between fractions 5 and 7 and appeared in the same peak as viral DNA (Fig. 7B, lower), indicating that HBV RNA is incorporated in the same viral particles as viral DNA. Therefore, circulating HBV RNA may be localized within CACs and/or virions.

To better characterize HBV RNA in CACs and virions, plasma sample from patient 0 was centrifuged through a 20% sucrose cushion and pellets were fractionated in a homogenous CsCl solution (1.18 g/cm^3^) as previously described (8). However, possibly due to a tendency of capsid particles to aggregate and stick to the wall of the centrifugation tube and the low density of the initial CsCl solution (8, 40), only mature DNA species from virions were detected in densities ranging from 1.22 to 1.24 g/cm^3^ (Fig. 7C, upper). Northern blot analyses demonstrated that the lengths of virion-associated HBV RNAs were approximately several hundred nucleotides (Fig. 7C, lower). Virion-associated RNAs were unlikely to be contaminated by CAC-associated HBV RNAs, since the immature SS DNA could not be observed even after a long exposure of X ray film. Moreover, RNA molecules would have been longer if there were CAC contamination (Fig. 7D, lower). Viral nucleic acids in pellets recovered from the centrifugation tube sidewalls could be readily detected on Northern (Fig. 7C, lower, lane P) or Southern (Fig. 7C, upper, lane P) blots using plus-strand-specific rather than minus-strand-specific riboprobe.

To analyze viral nucleic acids in CACs, concentrated plasma sample was separated in a higher CsCl density gradient (1.18 g/cm^3^ and 1.25 g/cm^3^). Both mature and immature viral DNA species were only detected in fractions with densities from 1.21 to 1.26 g/cm^3^ (Fig. 7D, upper), indicating the presence of a mixture of HBV virions and CACs. Viral RNAs were detected and ranged in length from a little shorter than the full-length pgRNA to a few hundred nucleotides (Fig. 7D, lower). Compared to virion-associated RNAs (Fig. 7C, lower), HBV RNA species detected in the mixture of CACs and virions were longer, with the longer RNA molecules possibly being associated with CACs.

### Extracellular HBV RNAs could serve as templates for synthesis of viral DNA.

**FIG 8:**
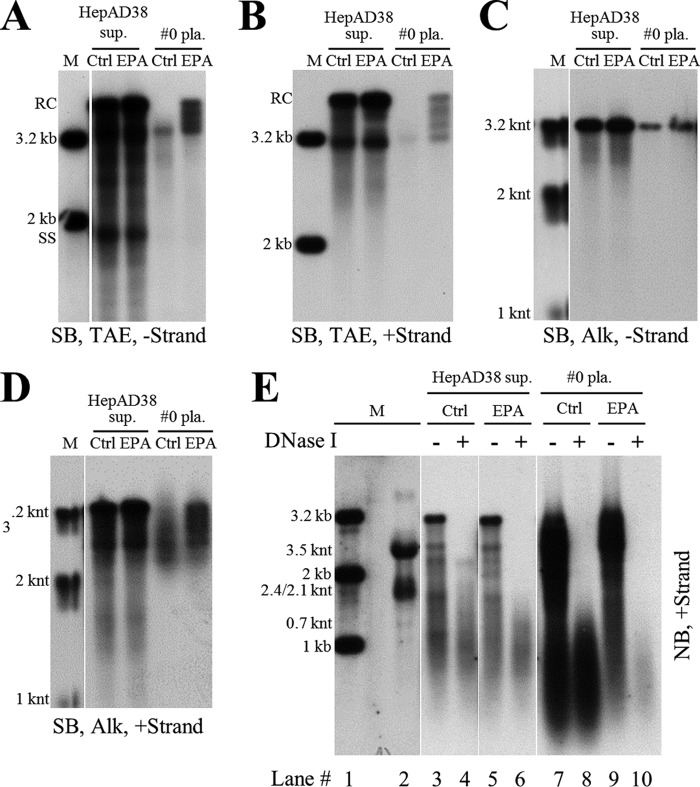
Analysis of extracellular HBV DNA and RNA by EPA. (A to D) Southern blot analysis of viral DNA strand elongation after EPA treatment. EPA was carried out employing HepAD38 cell culture supernatant and plasma sample from patient 0. Total nucleic acids were extracted via the SDS-proteinase K method. Viral DNA was separated by electrophoresis in TAE or alkaline agarose gels, followed by Southern blot analysis with minus- or plus-strand-specific riboprobes. (E) Northern blot analysis of viral RNA changed upon EPA treatment. Total viral nucleic acids (lanes 3, 5, 7, and 9) or RNA (treated with DNase I) (lanes 4, 6, 8, and 10) were separated by formaldehyde-MOPS agarose gel electrophoresis and subjected to Northern blotting.

Intracellular NCs are known to contain viral nucleic acids in all steps of DHBV DNA synthesis, including pgRNA, nascent minus-strand DNA, SS DNA, and RC DNA or DSL DNA (5). Our results showed that naked capsids contained almost the same DNA replicative intermediates as intracellular NCs (Fig. 1B and 2B) (7, 11). We also demonstrated that extracellular HBV RNAs within the naked capsids, CACs, and virions were heterogeneous in length (Fig. 1B, lower, 2F, and 7C and D). In the presence of deoxynucleoside triphosphates (dNTPs), viral RNA could be degraded and reverse transcribed into minus-strand DNA by the endogenous polymerase *in vitro* (5, 41, 42). Also, incomplete plus-strand DNA with a gap of about 600 to 2,100 bases could be extended by endogenous polymerase (43, 44). Based on these results, we wished to examine whether extracellular HBV RNAs could serve as RNA templates for viral DNA synthesis and be degraded by polymerase in the process. As shown in Fig. 8, endogenous polymerase assay (EPA) treatment of extracellular viral particles from either culture supernatant of HepAD38 cells or plasma sample from patients led to DNA minus (Fig. 8A and C)- and plus (Fig. 8B and D)-strand extension and, more importantly, HBV RNA signal reduction (Fig. 8E, lane 4 versus 6 and lane 8 versus 10). The apparent low efficiency of EPA reaction might have been due to our hybridization method, which detected both extended and unextended DNA strands rather than detecting only newly extended DNA.

In the process of HBV DNA replication, prior to minus-strand DNA synthesis, capsid-associated RNA is the full-length pgRNA. Upon transfer of viral polymerase-DNA primer to the 3′ DR1 region of pgRNA and cleavage of the 3′ epsilon loop RNA (a 3.2-knt pgRNA fragment remained), minus-strand DNA synthesis initiates and the pgRNA template is continuously cleaved from 3′ to 5′ by RNase H activity of viral polymerase. Consequently, from the initiation to the completion of minus-strand DNA synthesis, there will be a series of pgRNA fragments with receding 3′ ends ranging from 3.2 knt to 18 nt of the 5′ cap RNA primer (2, 21–24), representing the RNA templates that have not yet been reverse transcribed into minus-strand DNA. In addition to pgRNA with receding 3′ ends, there are also short RNA fragments arising from intermittent nicks by the RNase H domain of polymerase. Therefore, we used RNA probes spanning the HBV genome to map whether these RNA molecules are present in extracellular naked capsids and virions.

**FIG 9:**
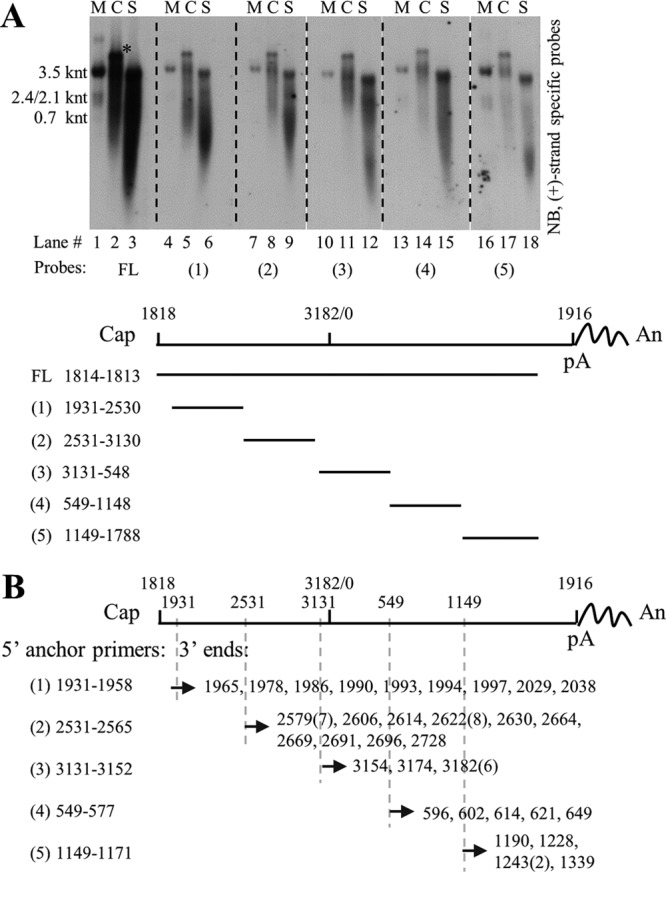
Mapping and identifying 3′ ends of extracellular HBV RNAs. (A) Northern blot detection of extracellular HBV RNAs with various riboprobes. Viral RNA from cytoplasmic (C) nucleocapsids (lanes 2, 5, 8, 11, 14, and 17) or culture supernatant (S) (lanes 3, 6, 9, 12, 15, and 18) of HepAD38 cells was extracted with TRIzol reagent and treated with DNase I before Northern blot analysis with plus-strand-specific riboprobes spanning the HBV genome as indicated. pgRNA was used as a reference, and map coordinates were numbered according to the sequence of the HBV genome (genotype D, accession number AJ344117.1). (B) Identification of 3′ ends of extracellular HBV RNAs. 3′ Ends of extracellular HBV RNAs were identified by the 3′ RACE method using different HBV-specific anchor primers (the same 5′ primers used for generating templates for producing riboprobes used in panel A, lower). Identified 3′ ends were numbered as described above, and numbers in parentheses indicate the amount of clones with the same 3′ ends. The asterisk indicates unknown nucleic acid copurified with intracellular capsid-associated viral RNA by TRIzol reagent. FL, full-length; Cap, 5′ cap of pregenomic RNA; pA, the polyadenylation site; An, poly(A) tail.

Five probes that spanned the HBV genome, except for the overlapping region between the 5′ end of pgRNA and the RNA cleavage site (nt 1818 to 1930), were prepared to map the extracellular HBV RNAs from HepAD38 cell culture supernatant (Fig. 9A). Intracellular nucleocapsid-associated HBV RNA from HepAD38 cells was used as a reference. As the probes moved from the 5′ end to 3′ end of pgRNA, especially for probes 1 to 4, RNA bands shifted from a wider range, including both short and long RNA species, to a narrower range, close to full-length pgRNA, with fewer RNA species detected (Fig. 9A, upper, lanes 2, 5, 8, 11, 14, and 17). Similarly, with the probes moving from the 5′ end to the 3′ end of pgRNA, a stronger intensity band representing extracellular HBV RNAs detected by each probe, especially for probes 1 to 4, was also shifting toward a longer RNA migration region (Fig. 9A, upper, lanes 3, 6, 9, 12, 15, and 18). It should be noted that the shifting pattern was more apparent when RNAs were detected with probes 1 to 4 but not with probe 5. It is possible that the reverse transcription speed is relatively quicker in the initial step (from the 3′ end of pgRNA, which overlaps the probe 5 sequence), and as a result, fewer pgRNA fragments will harbor RNA sequence for probe 5. Also, a short RNA species from either intracellular nucelocapsids or naked capsids and virions migrated faster than 0.7 knt and could be detected by all probes (Fig. 9A, upper, lanes 2, 3, 5, 6, 8, 9, 11, 12, 14, 15, 17, and 18). These RNA molecules likely represent the pgRNA fragments that have been hydrolyzed by the RNase H domain of viral polymerase (including the 3′ epsilon loop RNA cleaved by polymerase in the reverse transcription step) (24). Collectively, as predicted, longer extracellular HBV RNA species that migrated slower and closer to the position of pgRNA had longer 3′ ends, the shorter viral RNA molecules that migrated faster had relatively shorter 3′ ends, and the RNA species detected by all probes may represent products of pgRNA hydrolysis.

These results were further confirmed by employing a 3′ rapid amplification of cDNA ends (RACE) method. Various 3′ ends spanning the HBV genome were identified (Fig. 9B), validating the presence of 3′ receding RNA and the heterogeneous nature of extracellular HBV RNAs.

EPA treatment clearly demonstrated that extracellular HBV RNAs could be used as templates for DNA synthesis, and the presence of 3′ receding-end pgRNA fragments further confirmed not only the existence but also the use of such molecules as templates for viral DNA synthesis. Therefore, just like the viral RNA counterpart within intracellular NCs, extracellular HBV RNA molecules represent the RNA molecules generated in the process of viral DNA replication.

### ETV reduces viral DNA level but increases extracellular HBV RNA level in naked capsids and virions *in vitro*.

Entecavir (ETV), widely used in anti-HBV therapy, is a deoxyguanosine analog that blocks the reverse transcription and plus-strand DNA synthesis steps in the HBV DNA replication process (45–47). Treatment of CHB patients with nucleos(t)ide analogs (NAs), including entecavir, efficiently reduces the level of serum viral DNA but at the same time increases circulating HBV RNA levels (28, 48–52). We examined the effect of entecavir on the levels of both intracellular and extracellular viral nucleic acids in HepAD38 cell culture.

**FIG 10:**
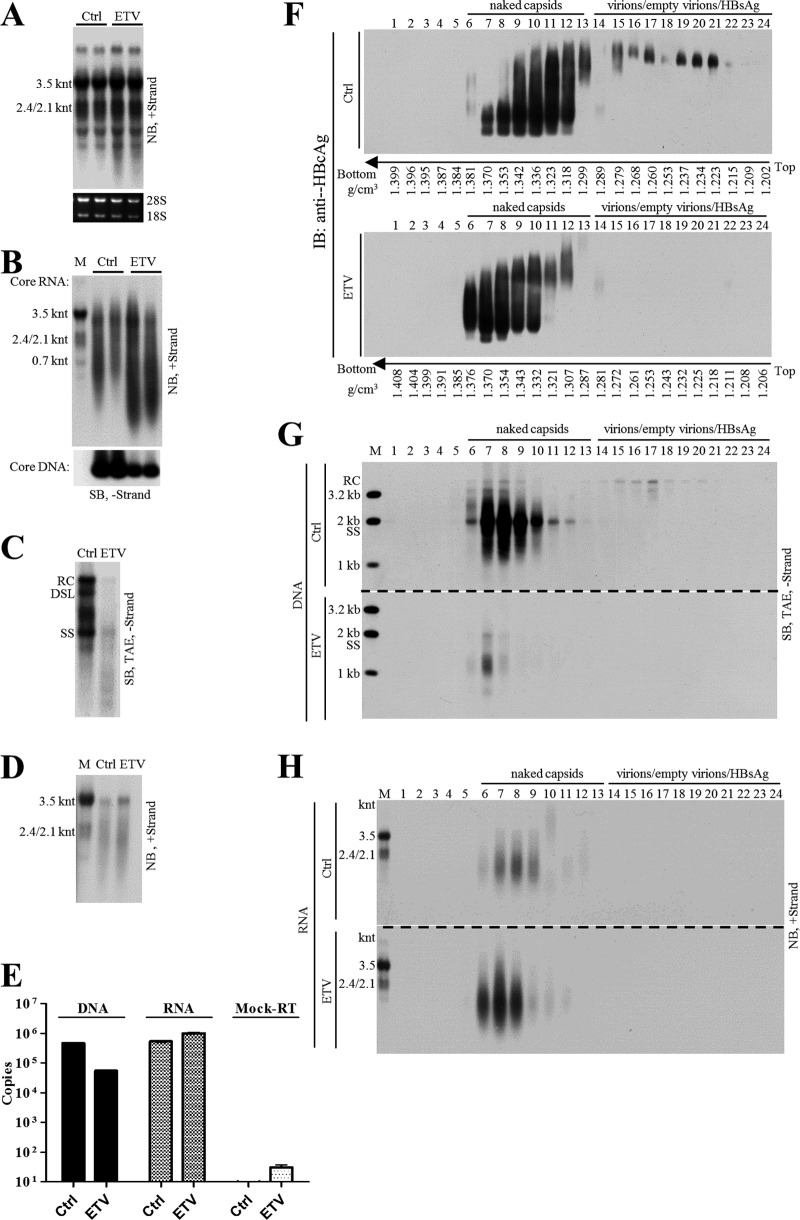
Analysis of HBV DNA and RNA change upon entecavir treatment of HepAD38 cells. (A) Change of total cellular HBV RNA level upon entecavir (ETV) treatment. HepAD38 cells were treated with ETV (0.1 μM) for 4 days, and total cellular RNA was analyzed by Northern blotting with ribosomal RNAs serving as loading controls. (B) Change of intracellular nucleocapsid-associated viral RNA (core RNA) and DNA (core DNA) level after ETV treatment. Cytoplasmic core RNA was extracted by the SDS-proteinase K method and analyzed by Northern blotting. Intracellular nucleocapsids were first separated by native agarose gel electrophoresis, and capsid-associated viral DNA (core DNA) was then probed with minus-strand-specific riboprobe. (C to E) Change of extracellular HBV DNA and RNA level upon ETV treatment. Total nucleic acids in HepAD38 cell culture supernatant were extracted and subjected to Southern and Northern blot analyses with specific riboprobes or quantification by PCR. (F to H) CsCl density gradient analysis of viral DNA/RNA level in naked capsids and virions after ETV treatment. HepAD38 cells were left untreated or were treated with ETV, and culture media were concentrated by ultrafiltration, followed by fractionation in CsCl density gradients as described in the legend to Fig. 4. Viral particles in each fraction were separated by native agarose gel electrophoresis, followed by immunoblotting with anti-HBcAg antibody. Viral DNA and RNA were extracted and subjected to Southern or Northern blot analyses.

Total viral RNA level remained unchanged or marginally increased upon entecavir treatment (Fig. 10A), and the intracellular capsid-associated viral RNA level was increased (Fig. 10B, upper). In contrast and as expected, the intracellular capsid-associated viral DNA level was decreased (Fig. 10B, lower). Similarly, extracellular viral DNA synthesis was significantly inhibited, while viral RNA was increased (Fig. 10C and D). Quantitative results showed that entecavir suppressed extracellular viral DNA to about one-tenth but at the same time increased viral RNA by about twofold the level for the untreated group (Fig. 10E).

Since viral DNA and RNA were enclosed in both naked capsids and virions, CsCl density gradient was used to separate these particles and to further study the antiviral effect of entecavir. As shown in Fig. 10, DNA-containing naked capsids were detected in fractions 6 to 11 and virions in fractions 15 to 24 (Fig. 10F). Entecavir effectively reduced viral DNA (Fig. 10G, fractions 6 to 10 and 15 to 17; this was also seen in a longer exposure of Fig. 10G [not shown]) but increased viral RNA content mainly in naked capsids (Fig. 10H, fractions 6 to 9). Moreover, the increase in RNA content within naked capsids led to an increased density of naked capsids (Fig. 10F, fractions 6 and 11, lower, versus fractions 6 and 11, upper). Interestingly, entecavir seemed to reduce HBcAg signal within virions (i.e., empty virions) (Fig. 10F, fractions 15 to 21, upper, versus fractions 15 to 21, lower) while increasing the egress of naked capsids from HepAD38 cells (data not shown).

## DISCUSSION

The RNA molecules in either intracellular NCs or extracellular virions were reported more than three decades ago (5, 41, 42), and naked capsids were shown to carry pgRNA *in vitro* (9, 11). Recently, it was suggested that the extracellular or circulating HBV RNA could serve as a surrogate marker to evaluate the endpoint of hepatitis B treatment (27, 30, 48–53). With this in mind and to facilitate its application as a novel biomarker for viral persistence, we studied the origin and characteristics of extracellular HBV RNA.

In the present study, we extensively characterized extracellular HBV RNAs and demonstrated that extracellular HBV RNAs were mainly enclosed in naked capsids rather than complete virions in supernatant of HepAD38 cells (Fig. 1B and 2F). These RNAs were of heterogeneous lengths, ranging from full-length pgRNA (3.5 knt) to a few hundred nucleotides. Furthermore, circulating HBV RNAs, also heterogeneous in length, were detected in blood of hepatitis B patients (Fig. 3D and 7C and D). Interestingly, the detection of HBV RNAs coincided with the presence of immature HBV DNA (Fig. 3D and E). Isopycnic CsCl gradient ultracentrifugation of RNA positive serum samples exhibited a broad range of distribution of immature HBV DNA, which contrasted with the results obtained in HepAD38 cells (Fig. 2B versus 4B and E, 7A). For the first time, we provided convincing evidence that unenveloped capsids containing the full spectrum of HBV replication intermediates and RNA species that are heterogeneous in length could be detected in the circulation of chronic hepatitis B patients.

**FIG 11:**
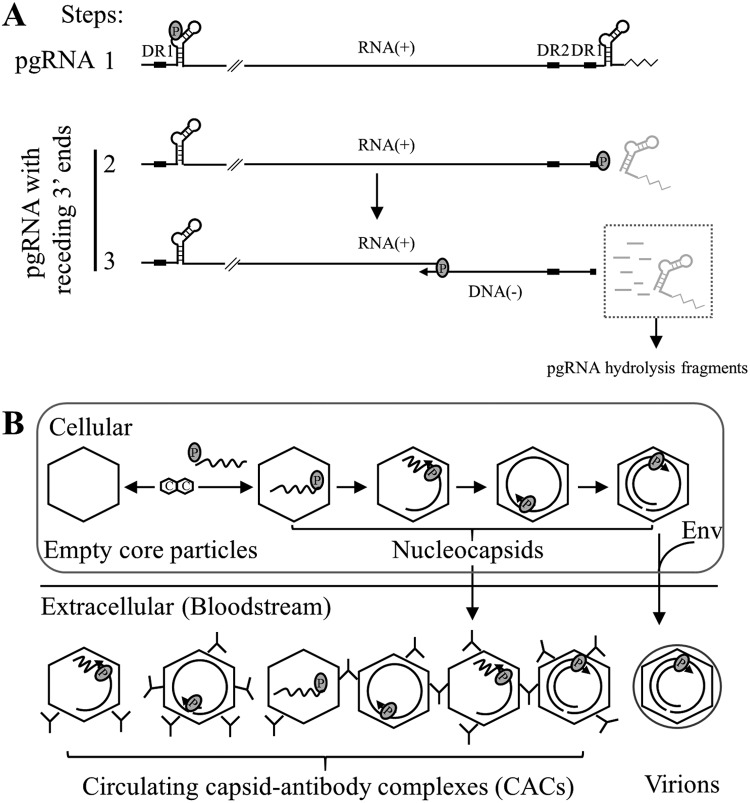
Models for the content of extracellular HBV RNAs and the formation of circulating CACs. (A) HBV RNA molecules present in the process of DNA synthesis. HBV RNAs are included in the following DNA synthesis steps: 1, encapsidation of full-length pgRNA into NCs; 2, transfer of polymerase-DNA primer to the 3′ DR1 region and initiation of minus-strand DNA synthesis (3′ epsilon loop of pgRNA will be cleaved by RNase H domain of polymerase); 3, elongation of minus-strand DNA. With the extension of minus-strand DNA, pgRNA will be continuously cleaved from the 3′ end, generating pgRNA fragments with receding 3′ ends and pgRNA hydrolysis fragments. (B) Possible forms of circulating CACs. Intracellular NCs with pgRNA or pgRNA fragment and DNA replicative intermediates released into blood circulation of CHB patients are bound with specific antibodies (IgG), forming various forms of CACs.

In view of our results and literature reports (2, 21–24), the presence of extracellular HBV RNAs could easily be interpreted in the context of the HBV DNA replication model (Fig. 11A). Since naked capsids contain viral DNA at all maturation levels, they will also carry HBV RNA molecules originating from pgRNA, including full-length pgRNA prior to minus-strand DNA synthesis, pgRNA with 3′ receding ends, and the pgRNA hydrolysis fragments. On the other hand, virions that contain only mature forms of viral DNA species would likely bear only the hydrolyzed short RNA fragments remaining in the nucleocapsid (43). Likewise, the HBV RNA species found in CACs are longer than those in virions in sera of hepatitis B patients (Fig. 7D, lower, versus C, lower). In line with this reasoning, treatment of HepAD38 cells with entecavir reduced viral DNA in naked capsids and virions (Fig. 10C, E, and G) but at the same time increased HBV RNA content within naked capsids (Fig. 10H). This may be a result of the stalled activity of viral RT with concomitant shutdown of RNA hydrolysis (46, 54).

Contrary to a recent report claiming that the pgRNA-containing NCs can be enveloped and secreted as virions (27), we clearly demonstrated that secreted naked capsids carry the majority of HBV RNAs (Fig. 1B and 2F) and that virion-associated RNAs are approximately several hundred nucleotides long (Fig. 1B and 7C). Our results are consistent with earlier reports demonstrating that only mature nucleocapsids with RC/DSL DNA are enveloped and secreted as virions (6–8, 11), and under this condition, virions carry only short RNase H-cleaved pgRNA (Fig. 11A, step 3).

In this research, we were unable to separate hydrolyzed pgRNA fragments from the pgRNA and pgRNA with 3′ receding ends. Thus, the length of these RNA molecules could not be determined. The existence of hydrolyzed RNA products during reverse transcription is not without precedent. In some retroviruses, DNA polymerization speed of RT is greater than the RNA hydrolysis speed of RNase H, thus hydrolysis of RNA template is often incomplete (55, 56). For example, RT of avian myeloblastosis virus (AMV) hydrolyzed RNA template once for every 100 to 200 nt, while cleavage frequency of RTs of human immunodeficiency virus type 1 (HIV-1) and Moloney murine leukemia virus (MoMLV) appeared to be around 100 to 120 nt (57). Moreover, RNA secondary structures, such as hairpins, may stall the RT activity promoting RNase H cleavage, producing shorter RNA fragments (55, 56).

Furthermore, the cleaved RNA fragments may not disassociate but anneal to the nascent minus-strand DNA forming the DNA-RNA hybrids until they are displaced by plus-strand DNA synthesis (55, 56). Although similar studies on HBV replication were hampered by lack of fully functional viral polymerase *in vitro* (58–61), the reported presence of DNA-RNA hybrid molecules clearly indicated the existence of degraded pgRNA fragments that still annealed to the minus-strand DNA (5, 41, 42, 62). Consistent with a previous study, our results also showed that at least part of the SS DNA is associated with RNA molecules as the DNA-RNA hybrid molecules, as detected by either RNase H digestion or the cesium sulfate density gradient separation method (5 and data not shown).

Given the fact that HBV RNA and immature HBV DNA are packaged in naked capsids (Fig. 1B and 2B and F) (11), we postulated that, in CHB patients, unenveloped capsids are released into circulation, where they rapidly form CACs with anti-HBcAg antibodies (Fig. 11B) (25, 33, 34). In support of this notion, we showed that protein A/G agarose beads could specifically pull down particles with mature and immature HBV DNA from sera of CHB patients, implying the involvement of antibody. Addition of anti-HBcAg antibody to HepAD38 cell culture supernatant led to a shift of naked capsids’ buoyant density to lower-density regions (Fig. 4C and D), a pattern similar to that obtained in HBV RNA-positive serum samples (Fig. 4B and E, and 7A). These particles exhibited heterogeneous electrophoretic behavior that differed from that of particles in HepAD38 culture supernatant, suggesting that they are not individual naked capsid particles but are associated with antibodies and have nonuniform compositions (Fig. 6 and 11B) (36–38). In CHB patients, the high titers of anti-HBcAg antibodies, which exceed 10,000 IU/ml, preclude circulation of antibody-unbound naked capsids (63). Indeed, the excessive amounts of anti-HBcAg antibodies present in the plasma sample of patient 0 were able to pull down naked capsids from the culture supernatant of HepAD38 cells (not shown).

We have demonstrated the presence of circulating CACs as the new form of naked capsids in CHB patients. It is known that naked capsid particles can be secreted either by the natural endosomal sorting complex required for transport (ESCRT) pathway (15–17) or possibly by cell lysis consequent to liver inflammation. Our preliminary clinical data (not shown) are in agreement with a recent study showing an association of circulating HBV RNA with serum ALT level (64). However, this connection can be interpreted in a different manner, as the capsid-antibody complexes might constitute a danger signal triggering inflammation. Interestingly, the release of naked capsids seems to be an intrinsic property of hepadnaviruses preserved through evolution. Recent studies by Lauber et al. provided evidence as to the ancient origin of HBV descending from nonenveloped progenitors in fish, with their envelope protein gene emerging *de novo* much later (65). Thus, it is reasonable to propose that the active release of HBV capsid particles should be deemed a natural course of viral egress.

Apart from HBV particles, it was also reported that exosomes could serve as HBV DNA or RNA carriers (29, 66, 67). However, HBV DNA and RNA was detected in naked capsids or CACs and virion fractions rather than in lower-density regions where membrane vesicles like HBsAg particles (density of 1.18 g/cm^3^) and exosomes (density of 1.10 to 1.18 g/cm^3^) would likely settle (2, 27, 48, 68, 69) (Fig. 1 and 7B). As a result, it is not likely that exosomes serve as the main vehicles carrying HBV DNA or RNA molecules.

Numerous pieces of data showed that HBV spliced RNAs also represent a species of extracellular HBV RNAs (28, 70, 71). However, in HepAD38 cells, as most of the RNAs are transcribed from the integrated HBV sequence other than the cccDNA template, pgRNA packaged into nucleocapsids is the predominant RNA molecule (Fig. 9A and 10D), and viral DNA derived from pgRNA is the dominant DNA form (Fig. 2D and E and data not shown). For the same reason, it would be difficult for us to estimate the amount of spliced HBV RNAs in clinical samples.

Although we could not completely rule out the possibility that HBV RNAs are released into blood circulation by association with other vehicles or other pathways, it is possible that the spliced HBV RNAs also egress out of cells in naked capsids and virions like the pgRNA.

In summary, we demonstrated that extracellular HBV RNA molecules are pgRNA and degraded pgRNA fragments generated in the HBV replication process *in vitro*. Moreover, we provided evidence that HBV RNAs exist in the form of CACs in hepatitis B patients’ blood circulation. More importantly, the association of circulating HBV RNAs with CACs or virions in hepatitis B patients suggests their pgRNA origin. Hence, our results here suggest the circulating HBV RNAs within CACs or virions in hepatitis B patients could serve as novel biomarkers to assess efficacy of treatment.

## MATERIALS AND METHODS

### Cell culture.

HepAD38 cells that replicate HBV in a tetracycline-repressible manner were maintained in Dulbecco’s modified Eagle’s medium (DMEM)-F12 medium supplemented with 10% fetal bovine serum, and doxycycline was withdrawn to allow virus replication (31).

### Patients and samples.

Serum samples from 45 chronic hepatitis B patients with HBV DNA titer higher than 10^7^ IU per ml were randomly selected. Detailed medical records of these patients are included in Table 1.

**TABLE 1:** Medical records of hepatitis B patients used in this research^*a*^

Patient no.	Sex	Age (yr)	HBV DNA titer (IU/ml)	HBeAg (IU/ml)	HBsAg (IU/ml)	ALT (IU/liter)	SS DNA result
0	NA	NA	2.67E + 06		4,932	396	+
1	M	54	1.24E + 07	25	>250	69	+
2	F	32	1.20E + 07	1,067	69,384	38	+
3	F	21	1.36E + 07	1,712	200	149	+
4	M	33	>5.00E + 07	4,812	113,933	133	+
5	NA	NA	1.25E + 07		3,423	33	−
6	M	26	1.17E + 07	545	2,759	22	−
7	M	36	1.77E + 07	4,332	19,541	136	**+**
8	M	35	>5.00E + 07	1,199	>250	104	**+**
9	M	26	2.20E + 07		>250	143	−
10	M	30	>5.00E + 07	2	4,265	123	−
11	F	23	>5.00E + 07	20	5,757	120	**+**
12	M	37	2.07E + 07	2,315	16,128	177	**+**
13	M	28	>5.00E + 07	3,495	60,676	58	NA
14	F	28	>5.00E + 07	16,515	89,575	78	+
15	M	37	1.62E + 07	574	+, ND	112	+
16	M	NA	>5.00E + 07	1,601	>250	22	NA
17	M	15	2.28E + 07	2,038	32,739	180	+
18	M	41	2.71E + 07	694	>250	313	+
19	M	34	2.35E + 07	80	32,514	148	+
20	F	44	>5.00E + 07	1,596	4,306	172	−
21	M	NA	3.48E + 07	107	>250	103	+
22	NA	NA	>5.00E + 07	2024	45,873	147	+
23	M	20	1.32E + 07	13,411	12,387	344	+
24	M	48	>5.00E + 07	5,511	76,914	33	−
25	M	NA	3.15E + 07		15,984	366	−
26	M	31	4.16E + 07	10,251	50,469	442	+
27	M	60	1.35E + 07	749	>250	105	+
28	F	41	>5.00E + 07	4,173	>52,000	194	+
29	NA	NA	>5.00E + 07	4,233	49,125	39	+
30	M	29	1.42E + 07	25	5,800	940	+
31	M	27	2.34E + 07	1,117	22,412	129	+
32	M	37	2.65E + 07		70	109	NA
33	NA	NA	2.03E + 07		4,902	111	+
34	M	32	>5.00E + 07	993	43,582	249	+
35	NA	NA	2.94E + 07	4,641	93,336	12	+
36	NA	NA	>5.00E + 07	10,956	2,496	108	+
37	F	43	>5.00E + 07	1,021	>250	74	+
38	F	28	>5.00E + 07	215	446	26	+
39	M	31	>5.00E + 07	+, ND	38,165	194	+
40	NA	NA	>5.00E + 07	25	>250	69	+
41	M	26	1.52E + 07	+, ND	+, ND	95	+
42	M	25	>5.00E + 07	6,300	43,151	373	+
43	M	22	>5.00E + 07	3,844	23,620	329	+
44	M	27	1.36E + 07	1,185	11,106	149	+
45	M	44	1.28E + 07	663	23,330	425	−
46	F	29	>5.00E + 07	+, ND	+, ND	667	+

a NA, not available; ND, not determined; M, male; F, female; sera from patients 0 and 46 were not included with sera from other patients for SS DNA screening.

Plasma sample was the plasma exchange product obtained from an HBeAg-negative hepatitis B patient (patient 0) (HBV genotype B with A1762T, G1764A, and G1869A mutation) who died of fulminant hepatitis as a consequence of reactivation of hepatitis B (Table 1).

### Ethics statement.

All samples from HBV-infected patients used in this study were from an already-existing collection supported by the National Science and Technology Major Project of China (grant no. 2012ZX10002007-001). Written informed consent was received from participants prior to collection of clinical samples (72). Samples used in this study were anonymized before analysis. This study was conducted in compliance with the ethical guidelines of the 1975 Declaration of Helsinki and was approved by the ethics committee of the Shanghai Public Health Clinical Center.

### Preparation of viral particles.

HepAD38 cell culture supernatant was mixed with polyethylene glycol 8000 (PEG 8000) to a final concentration of 10% (wt/vol) and incubated on ice for at least 1 h, followed by centrifugation at 925 × *g* for 20 min. Pellets were suspended in TNE buffer (10 mM Tris-Cl [pH 7.5], 100 mM NaCl, and 1 mM EDTA) containing 0.05% β-mercaptoethanol to 1/150 of the original volume, followed by a brief sonication (73, 74). Alternatively, viral particles in HepAD38 cell culture supernatant were concentrated 50- to 100-fold by ultrafiltration using a filter unit (Amicon Ultra-15, 100 kDa).

Plasma samples from patient 0 were centrifuged through a 20% (wt/vol) sucrose cushion at 26,000 rpm for 16 h in an SW 32 Ti rotor (Beckman), and pellets were resuspended in 1/200 the original volume of TNE buffer and sonicated briefly (75).

Samples prepared using methods described above were either used immediately or aliquoted and stored at −80°C for later use.

### Sucrose density gradient centrifugation.

HepAD38 cells culture supernatant concentrated by PEG 8000 was centrifugation at 500 × *g* for 5 min to remove aggregates. Ten percent, 20%, 30%, 40%, 50%, and 60% (wt/wt) sucrose gradients were prepared by underlayering and incubated for 4 h in a water bath at room temperature to allow gradient to become continuous. Five hundred microliters of concentrated sample was layered over the gradient and centrifuged at 34,100 rpm for 14 h at 4°C in a Beckman SW 41 Ti rotor. Fractions were collected from top to bottom, and the density of each fraction was determined by refractometry (10). Fractions containing viral particles were subjected to native agarose gel analysis, and HBsAg level was determined by enzyme-linked immunosorbent assay (ELISA) (Shanghai Kehua).

### Cesium chloride density gradient centrifugation.

HepAD38 cell culture supernatant (1.5 ml), concentrated by ultrafiltration, or serum samples from chronic hepatitis patients diluted with TNE buffer to 1.5 ml were mixed with equal volumes of 37% (wt/wt) CsCl-TNE buffer (1.377 g/cm^3^) and underlayered with 1.9 ml 34% (wt/wt) CsCl-TNE buffer (1.336 g/cm^3^), followed by centrifugation at 90,000 rpm at 4°C for 12 h (Beckman VTi 90 rotor) (8). The tube was punctured from the bottom, and every six to seven drops were collected as one fraction. Densities of separated fractions were determined by weighing. Each fraction was then desalted against TNE buffer by ultrafiltration, followed by native agarose gel separation or nucleic acid extraction.

All of the CsCl density gradient centrifugation experiments were carried out at 90,000 rpm at 4°C for 12 h in a Beckman VTi 90 rotor.

### Native agarose gel analysis of viral particles and capsid-associated DNA.

Viral particles were resolved by native agarose gel (0.8% agarose gel prepared in Tris-acetate-EDTA [TAE] buffer) electrophoresis and transferred in TNE buffer to either a nitrocellulose membrane (0.45 μM) for detection of viral antigens with specific antibodies or a nylon membrane for Southern blot analysis of viral DNA. For viral antigens detection, the membrane was first fixed as previously described (74), and HBV core antigen was detected by anti-HBcAg antibody (Dako) (1:5,000). The same membrane then was soaked in stripping buffer (200 mM glycine, 0.1% SDS, 1% Tween 20, pH 2.2) and reprobed with anti-HBsAg antibody (Shanghai Kehua) (1:5,000). For Southern blot analysis of viral DNA, the membrane was dipped in denaturing buffer (0.5 N NaOH, 1.5 M NaCl) for 10 s and immediately neutralized in 1 M Tris-Cl (pH 7.0)–1.5 M NaCl for 1 min, followed by hybridization with minus-strand-specific riboprobe (76).

### Viral nucleic acid extraction, separation, and detection.

**(I) Nucleic acid extraction.** To extract total viral nucleic acids (DNA and RNA), the SDS-proteinase K method was used (77). Samples were digested in solution containing 1% SDS, 15 mM EDTA, and 0.5 mg/ml proteinase K at 37°C for 15 min. The digestion mixture was extracted twice with phenol and once with chloroform. Aqueous supernatant were added with 1/9 volume of 3 M sodium acetate (pH 5.2) and 40 μg of glycogen and precipitated with 2.5 volumes of ethanol.

In addition to the SDS-proteinase K method, viral RNA was also extracted with TRIzol LS reagent according to the manufacturer’s instructions (Thermo Fisher Scientific).

To isolate intracellular capsid-associated viral RNA, HepAD38 cells were lysed in NP-40 lysis buffer (50 mM Tris-Cl [pH 7.8], 1 mM EDTA, 1% NP-40), and cytoplasmic lysates were incubated with CaCl_2_ (final concentration, 5 mM) and micrococcal nuclease (MNase) (Roche) (final concentration, 15 U/ml) at 37°C for 1 h to remove nucleic acids outside nucleocapsids. The reaction was terminated by addition of EDTA (final concentration, 15 mM), and then proteinase K (0.5 mg/ml without SDS) was added to the mixture, followed by incubation at 37°C for 30 min to inactivate MNase. Viral nucleic acids were released by addition of SDS to a final concentration of 1% and extracted as described above.

**II. Separation. (i) TAE agarose gel.** Viral DNA was resolved by electrophoresis through a 1.5% agarose gel in 1× TAE buffer, followed by denaturation in 0.5 M NaOH–1.5 M NaCl for 30 min and neutralization with 1 M Tris-Cl (pH 7.0)–1.5 M NaCl for 30 min.

**(ii) Alkaline agarose gel.** Viral DNA was denatured with a 0.1 volume of solution containing 0.5 M NaOH and 10 mM EDTA and resolved overnight at 1.5 V/cm in a 1.5% agarose gel with 50 mM NaOH and 1 mM EDTA. After electrophoresis, the gel was neutralized with 1 M Tris-Cl (pH 7.0)–1.5 M NaCl for 45 min (78).

**(iii) Formaldehyde-MOPS agarose gel.** Viral RNA was obtained by treatment of total nucleic acids extracted using the above-described SDS-proteinase K method with RNase free DNase I (Roche) for 15 min at 37°C. The reaction was stopped by addition of equal amounts of 2× RNA loading buffer (95% formamide, 0.025% SDS, 0.025% bromophenol blue, 0.025% xylene cyanol FF, and 1 mM EDTA) supplemented with extra EDTA (20 mM), followed by denaturing at 65°C for 10 min. Viral RNA extracted by TRIzol LS reagent was mixed with 2× RNA loading buffer and denatured. Denatured mixtures were separated by electrophoresis through a 1.5% agarose gel containing 2% (vol/vol) formaldehyde solution (37%) and 1× MOPS (3-[N-morpholino]propanesulfonic acid) buffer.

The gels described above were balanced in 20× SSC solution (1× SSC is 0.15 M NaCl and 0.015 M sodium citrate, pH 7.0) for 20 min, and viral nucleic acids were transferred onto nylon membranes overnight with 20× SSC buffer.

### III. Detection.

Digoxigenin-labeled riboprobes used for detection of HBV DNA and RNA were prepared by *in vitro* transcription of a pcDNA3 plasmid that harbors 3,215 bp of HBV DNA (nt 1814 to 1813) by following the vendor’s suggestions (12039672910; Roche). Riboprobes used for HBV RNA mapping were transcribed from DNA templates generated by PCR by incorporating T7 promoter into the 5′ end of reversed primers (Fig. 9A).

Hybridization was carried out at 50°C overnight, followed by two 5-min washes in 2× SSC–0.1% SDS at room temperature and two additional 15-min washes in 0.1× SSC–0.1% SDS at 50°C. The membrane was sequentially incubated with blocking buffer and anti-digoxigenin-AP Fab fragment (Roche) at 20°C for 30 min. Subsequently, the membrane was washed twice with washing buffer (100 mM maleic acid, 150 mM NaCl, and 0.3% Tween 20, pH 7.5) for 15 min, followed by detection with diluted CDP-Star substrate (ABI) and exposure to X-ray film.

### Protein A/G agarose bead pulldown of antibody-antigen complexes.

Two hundred microliters of serum sample was first mixed with 300 μl of TNE buffer, and then 15 μl of protein A/G agarose bead slurry (Santa Cruz) was added to the mixture, followed by incubation overnight at 4°C in a sample mixer. Subsequently, protein A/G agarose beads were washed three times with TNE buffer, and viral DNA in input serum samples (40 μl) and agarose bead pulldown mixtures were extracted and subjected to Southern blot analysis.

### EM.

Serum samples from patients 11, 17, 21 22, 23, 27, 28, 30, and 41 were pooled (200 μl each) and mixed with 200 μl of 20% (wt/wt) sucrose. Serum mixtures were centrifuged through 2 ml of 20% (wt/wt) and 2 ml of 45% (wt/wt) (1.203 g/cm^3^) sucrose cushions at 34,100 rpm for 8 h at 4°C in an SW 41 Ti rotor (Beckman) to remove HBsAg particles. Supernatants were decanted and the centrifugation tube was placed upside down for 20 s, and residue sucrose was wiped out. One milliliter of phosphate buffer (10 mM Na_2_HPO_4_, 1.8 mM KH_2_PO_4_, and no NaCl) (pH 7.4) was added, and the bottom of the tube was gently washed without disturbing the pellet. A volume of 11.5 ml of phosphate buffer then was added into the tube and centrifuged again at 34,100 rpm for 3 h at 4°C. The pellet was resuspended in a drop of distilled water and dropped onto a carbon-coated copper grid, followed by staining with 2% phosphotungstic acid (pH 6.1) and examining in an electron microscope (Philip CM120) (13, 79).

### Viral DNA and RNA quantification.

Viral DNA used for quantification was extracted using the SDS-proteinase K method as described above. Viral RNAs were extracted by TRIzol LS reagent, and DNase I was used to remove the remaining DNA, followed by phenol and chloroform extraction and ethanol precipitation. Reverse transcription was carried out using Maxima H minus reverse transcriptase (Thermo Fisher Scientific) with a specific primer (AGATCTTCKGCGACGCGG [nt 2428 to 2411]) according to the manufacturer’s guidelines, except the 65°C incubation step was skipped to avoid RNA degradation. To ensure removal of viral DNA signal (below 1,000 copies per reaction), a mock reverse transcription, without addition of reverse transcriptase, was carried out. Quantitative real-time PCR (qPCR) was carried out using Thunderbird SYBR qPCR mix (Toyobo) in a StepOnePlus real-time PCR system (ABI). Primer pairs (F, GGRGTGTGGATTCGCAC [nt 2267 to 2283]; R, AGATCTTCKGCGACGCGG [nt 2428 to 2411]) conserved among all HBV genotypes and close to the 5′ end but not in the overlap region between the start codon and the poly(A) cleavage site of pgRNA were chosen. The cycling conditions were 95°C for 5 min, followed by 40 cycles of 95°C for 5 s, 57°C for 20 s, and 72°C for 30 s. DNA fragment containing 3,215 bp of full-length HBV DNA was released from plasmid by restriction enzymes, and DNA standards were prepared according to a formula in which 1 pg of DNA equals 3 × 10^5^ copies of viral DNA.

### EPA.

HepAD38 cell culture supernatant or plasma from patient 0 were concentrated as described above and mixed with equal volumes of 2× EPA buffer (100 mM Tris-Cl, pH 7.5, 80 mM NH_4_Cl, 40 mM MgCl_2_, 2% NP-40, and 0.6% β-mercaptoethanol) with or without dNTPs (dATP, dCTP, dGTP, and dTTP, each at a final concentration of 100 μM) (80). The reaction mixtures were incubated at 37°C for 2 h and stopped by addition of EDTA to a final concentration of 15 mM.

### 3′ RACE.

Concentrated HepAD38 cell culture supernatant (by ultrafiltration) was digested with MNase in the presence of NP-40 (final concentration, 1%) for 30 min at 37°C. EDTA (final concentration, 15 mM) and proteinase K (final concentration, 0.5 mg/ml) were then added and incubated for another 30 min at 37°C. Viral nucleic acids were extracted with TRIzol LS reagent followed by DNase I treatment to remove residue viral DNA. Poly(A) tails were added to the 3′ end of HBV RNA by E. coli poly(A) polymerase (NEB). The preincubation step at 65°C for 5 min was omitted to reduce potential RNA degradation, and reverse transcription was carried out with Maxima H minus reverse transcriptase (Thermo Scientific) using an oligo-dT(29)-SfiI(A)-adaptor primer (5′-AAGCAGTGGTATCAACGCAGAGTGGCCATTACGGCCTTTTTTTTTTTTTTTTTTTTTTTTTTTTT-3′) in reverse transcription buffer [1× RT buffer, RNase inhibitor, 1 M betanine, 0.5 mM each dNTP, and 5 μM of oligo-dT(29)-SfiI(A)-adaptor primer] at 50°C for 90 min, followed by heating at 85°C for 5 min and treatment with RNase H at 37°C for 15 min. PCR amplification of cDNA fragments was then performed with 5′ HBV-specific primers [the same sequences of forward primers used for riboprobe preparation (Fig. 9A), except each primer containing a flanking sequence plus a SfiI(B) site (5′-AGTGATGGCCGAGGCGGCC-3′)] and 3′ adaptor primer (5′-AAGCAGTGGTATCAACGCAGAGTG-3′). The reaction was carried out with PrimeSTAR HS DNA polymerase (TaKaRa) at 95°C for 5 min, followed by 5 cycles of 98°C for 5 s, 50°C for 10 s, and 72°C for 210 s, 35 cycles of 98°C for 5 s, 55°C for 10 s, and 72°C for 210 s, and a final extension step at 72°C for 10 min. PCR amplicons were digested with SfiI enzyme and cloned into pV1-Blasticidin vector (kind gift from Zhigang Yi, Shanghai Medical College, Fudan University). Positive clones were identified by sequencing, and only clones with 3′ poly(dA) sequence were considered authentic viral RNA 3′ ends.
